# Humidity Detection and Hygropreference Behavior in Larvae of the Tobacco hornworm, *Manduca sexta*

**DOI:** 10.1673/031.007.3901

**Published:** 2007-06-18

**Authors:** Marc Rowley, Frank Hanson

**Affiliations:** ^1^ Berea College - Biology, CPO 1972,101 Chestnut Street, Berea, KY 40404; ^2^ UMBC - Biological Sciences, 1000 Hilltop Circle, Baltimore, MD 21250

**Keywords:** hygroreceptor, hygroreception, water detection, relative humidity

## Abstract

Water is a critical resource for any terrestrial animal, especially for a soft-bodied insect such as larvae of the tobacco hornworm, *Manduca sexta* L. (Lepidoptera: Sphingidae). Strategies for coping with a dry environment might include seeking out regions of high relative humidity that reduce desiccative stress, or to find and imbibe liquid water. Desiccated larvae placed in a linear arena with a humidity gradient preferred the humid end, whereas un-desiccated larvae did not. This behavior was not affected by temperature. Ablation or occlusion of the antennae showed that they are required to mediate this behavior. A series of experiments showed that control larvae oriented towards and imbibed liquid water whereas those whose antennae had been occluded with wax did not. Electrophysiological recordings from the lateral basiconic sensillum of the second antennal segment revealed the presence of at least one hygroreceptive unit that greatly increased its firing rate in response to moist air, decreased firing rates in response to dry air, and showed mild post-stimulatory inhibition.

## Introduction

The sense organs of even relatively simple organisms such as arthropods detect a variety of important nutrients, water being probably the most critical for terrestrial animals. Since their own water potential normally exceeds that of their surroundings, there is a net water efflux which must be continually replenished by water from an external source because metabolic water is rarely sufficient.

The ability to detect water in the environment (hygroreception) has been documented in a variety of arthropods. Arachnids (Ehn & Tichy 1992, Kröber & Guerin 1999, Gaffin et al. 1992), Blattodea (Yokohari 1978; Yokohari & Tateda 1976; Dambach & Goehlen 1999), Coleoptera (Harbach & Larson 1977, Iwasaki et al. 1995), Collembola (Hayward et al. 2000), Diptera (Kellogg 1970), Hemiptera (Guarneri et al. 2002), Hymenoptera (Steidle & Reinhard 2003, Yokohari et al. 1982; Yokohari 1983), and adult Lepidoptera (Steinbrecht & Müller 1991) are all known to have water detecting sensory systems or related behavior.

These studies paint a general picture of hygroreception in arthropods. Typically, such behaviors are mediated by a sensillar structure that houses both wet and dry receptor neurons as well as a temperature receptor (Ehn & Tichy 1994, Iwasaki et al. 1995, Yokohari 1978, Yokohari 1983, Yokohari & Tateda 1976, Yokohari et al. 1982). The structure itself is a blind, pore-less sensilla innervated by only these three sensory neurons and their accessory cells (Iwasaki et al. 1995, Steinbrecht & Müller 1991, Yokohari 1983). It is thought that the hygroreceptive cells are modified mechanoreceptors and that they respond to changing volumes of some hygroscopic material contained within the sensilla (Steinbrecht & Müller 1991, Yokohari 1978). Such structures are usually located with other sensory structures on the antennae or tarsi of the animal.

When tested behaviorally, the animals mentioned above display locomotory or other decision-making behaviors based on moisture cues. Since small arthropods have an unfavorable surface area to volume ratio, it is likely that they experience considerable desiccative stresses, especially when exposed to the warm, arid environments that many of them inhabit. One of the simplest strategies an animal can employ to protect itself from water loss is to preferentially inhabit areas of higher relative humidity, using hygroreceptive neurons to identify regions of high humidity. Those arthropods that lack a thickened waxy cuticle may be most likely to employ this strategy.

As small, soft-bodied animals, caterpillars are at particular risk of desiccation when exposed to arid environments. Greenhouse observations indicate that larval *Manduca sexta* typically roost on the undersides of their host plant's leaves where the relative humidity is measurably higher than that of the upper leaf surface. Larvae feeding on exposed portions of the plant return to the underside of a leaf to molt. These observations suggest that the larvae may be using hygropreference behavior to protect themselves from water loss while roosting or molting.

Based on such observations, we hypothesize that larval *M. sexta* can detect humidity and use this information to make locomotory decisions for hydration homeostasis, as do the other arthropods previously mentioned. To test this hypothesis, the behavior of larval *M. sexta* in controlled environmental arenas was observed under conditions of differential desiccative stress and at various temperatures. Antennal occlusion and ablation experiments were also conducted to determine what role the antennae play in mediating this behavior.

The sensory basis of this behavior may be the neurons located in sensilla on the second antennal segment that respond to changes in relative humidity. These receptor cells respond only to moisture and do not occur in antagonistic pairs of moist-and-dry-sensitive cells as is typical of other insect humidity systems (Dethier and Schoonhoven 1968). To our knowledge, little else is known about the physiology of these humidity-detecting cells or the behavioral relevance of this information in caterpillars. Therefore, action potentials from these neurons were recorded electrophysiologically while stimulating with humid and dry air to verify this earlier report.

## Methods

### Rearing of Larvae

Larval *M. sexta* L. (Lepidoptera: Sphingidae) were raised from eggs obtained from the North Carolina State University insectary. Larvae were reared on artificial diet (Bioserv, www.bioserv.com) at room temperature under a 16:8 L:D cycle. At the beginning of the molt into the fifth larval stage, larvae were isolated from the culture and placed in compartmentalized plastic containers without food. After molting, larvae were examined under a dissecting microscope to ensure the physical integrity of their external sensory structures, and then placed into a treatment within one day post-molt.

**Figure 1.**
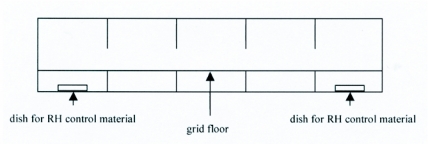
2-D Schematic side view of the hygropreference arena.

### Hygropreference behavior

#### Hygropreference arena

Linear behavioral arenas were constructed of one-quarter inch thick clear plastic and measured 61cm long × 15cm wide × 14cm high (Figure 1). The arena was partially divided into five equally sized chambers by vertical vanes attached to the bottom and top of the box. These partial dividers impeded air circulation and helped to maintain the humidity gradient while permitting the larva to move freely from chamber to chamber. The floor was a grid made of fine mesh screen supported by a vinyl coated, stiff, coarse wire mesh. The grid was elevated 3.8 cm above the bottom of the arena, supported by the lower vertical vanes. This floor provided the larva secure footing while preventing it from contacting the humidity-controlling materials below. The top of the arena had a one-inch diameter hole above the center of each chamber that permitted introduction of the larva or a temperature/humidity probe without disrupting the established humidity gradient. During experimentation these holes were covered with small plastic squares.

**Table 1:**
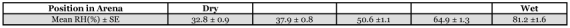
Arena humidity profile showing the average relative humidity ± SE in each chamber of the hygropreference arena (N = 65 trials)

Temperature within the arenas was maintained with resistive heating elements beneath the black painted steel plate on which the arenas were placed and controlled by a thermostat to within ± 1 °C. To prevent condensation on the walls, and to ensure even heating across the arenas, the entire setup was covered with a clear plastic lid and enclosed by one inch thick insulating foam blocks covered with black fabric (to absorb scattered light). Temperature and humidity were verified by sensors either introduced through the hole above each arena, or located beneath the grid floor. In either case the measurements were taken as close to the grid floor as possible to reflect the conditions that the larvae were exposed to.

#### Desiccation chambers

Experimental larvae were treated in a desiccation chamber constructed from a disposable Ziploc® box (square, 4 cup size, 13.7 × 13.7 × 8.8 cm). Within the box an elevated screen floor separated the larvae from the desiccating materials. Four larvae were treated in each chamber. Within each chamber, larvae were placed within individual tubes of fine mesh screen to prevent contact between larvae and permit individual identification.

#### Treatments

Within 24 hours after molting into their fifth larval stage, larvae were weighed and placed into desiccation chambers containing either silica gel (< 10% RH) for the desiccated group or nothing (≈ 65% RH) for the control group and incubated at 30 °C for 24 hours. Subsequently, the larvae were re-weighed. The desiccated larvae lost 20.5% (SE = 0.5, N = 133) and control larvae lost 4.6% (SE = 0.22, N = 119) of their body mass respectively.

**Figure 2.**
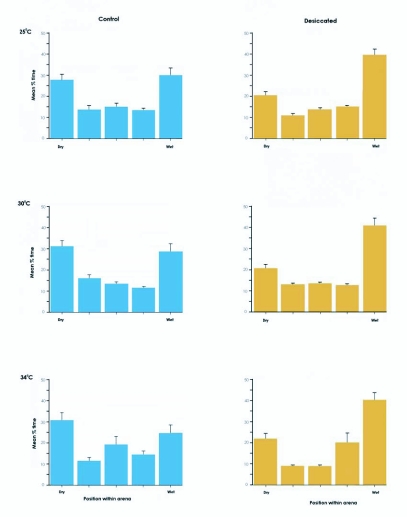
Behavior in the hygropreference arena. The y-axis represents the percentage of the total number of time-points that the animal spent in a particular chamber of the arena. The x-axis represents the location within the arena. Bars are means of 20 trials plotted with standard errors.

For the sensory occlusion experiments, occlusion of the antennae was performed after this desiccation treatment. Larvae were held under a dissecting microscope while a small heated wire loop was used to cover the antennae with commercial dental wax or a 1:1 mixture of beeswax and rosin. After behavioral trials, these larvae were inspected visually to ensure that the sensory structures had remained occluded throughout the trial.

For the antennal ablation experiments, ablations were performed prior to the desiccation regime. Larvae were anesthetized by submerging them in water for several minutes (Gothilf & Hanson, 1994). Larvae were then held under water in a small dish and their antennae were removed using a pair of iridectomy scissors. Larvae were then removed from the water and wiped dry before being weighed and placed into the desiccation chambers. By performing the surgical ablation before desiccation the larvae were permitted a 24 hour recovery period as they were undergoing desiccation treatment.

**Figure 3.**
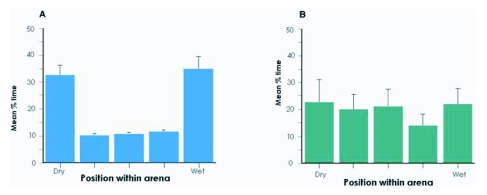
Antennal occlusion and ablation behavior at 30°C. (A) Desiccated larvae with their antennae covered with wax (N = 20). (B) Desiccated larvae from which the antennae were surgically removed (N = 10) Bars are means plotted with standard errors.

#### Arena setup

Prior to the beginning of each experiment the arenas were thoroughly cleaned with a bottle brush and ethanol. The humidity gradient was established by placing a 5 cm Petri dish of water at one end of the arena and a similar dish of silica gel at the other end (Table 1). The positions of water and silica gel were alternated every trial to prevent directional bias.

At the start of the trial, a single larva was introduced to the center chamber of each arena via the hole in its lid. After an acclimation period of at least fifteen minutes, the imaging software began taking images at a rate of one per minute for 60 minutes.

#### Data collection and analysis

The three behavior arenas were simultaneously monitored by a computer imaging system consisting of an Apple iSight camera, a Macintosh G4 computer, and Oculus webcam software. Lighting was provided by two fluorescent light strips placed outside plastic windows in the insulating foam at the level of the grid floor.

Images were first processed to enhance contrast using NIH's ImageJ software (available at http://rsb.info.nih.gov/ij/). Custom software written in MATLAB presented the viewer with each image and the user identified the location of the larva within the arena at each time-point. The raw data are simply counts of the number of time-points that the larva was observed in each chamber.

To analyze the effect of treatment, each set of experimental data was compared with its corresponding control set using a chi-squared test (Langley, 1970) on the raw counts from the image analysis.

#### Liquid water orientation experiments

Arenas were constructed by filling standard 5 cm Petri dishes with a small amount of melted paraffin wax. When hardened, the wax permitted the placement of an exact amount of water in a cohesive drop. Non-desiccated fifth instar larvae were tested either untreated (control) or with their antennae covered with a 1:1 beeswax-rosin mixture (occluded). Larvae were assigned to treatment groups and then placed in isolation chambers for at least 15 minutes post-treatment to permit recovery. The arenas were cleaned with ethanol prior to the start of each experiment. A single 0.1ml drop of purified water (BioWhittaker # 17-724Q, Cambrex BioScience) was placed in the middle of each arena, the animals were introduced, and the Petri dish was covered. Trials were observed for 30 minutes for contact and consumption of the water drop by the larvae.

**Figure 4.**
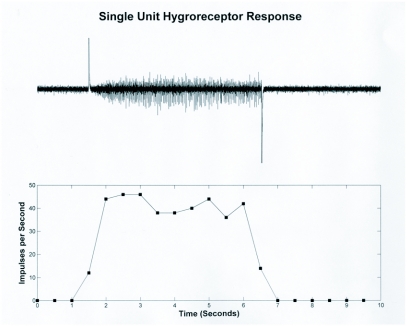
Single unit hygroreceptor response. The trace is a ten second recording from a single hygroreceptor unit (presumably a sensory neuron). The large artifacts in the trace indicate the switch from dry to moist air stimulus (positive going) and then back to dry air (negative going). The graph shows the activity of the unit in 0.5 second bins.

#### Electrophysiology

Electrophysiological recordings were made on a live larva preparation following the method of Gothilf and Hanson (1994). Briefly, a larva was anesthetized by submerging in water for several minutes. The larva was then placed into a glass vial filled with 0.1M KCl. The larva's head was placed through a hole in a latex membrane that was used with the vial's lid to seal the preparation. A silver wire was inserted through the wall of the vial into the KCl solution and served as the reference electrode.

The antenna was immobilized with a small amount of a 1:1 beeswax / rosin mixture placed at the base of the antenna using a heated wire loop, but without covering the sensory structures at the tip of the antenna. Contact of the heated loop with the antenna was avoided to minimize heat damage to the sensory neurons within.

Recordings were made using a sharpened tungsten wire electrode. The action potentials were amplified using a custom, high-impedance amplifier (George Johnson, UMBC) with a band pass of 50 Hz — 2 kHz. The data acquisition card sampled at 10 KHz under STA software (Eric Stadler, Wadenswil, Switzerland).

The stimulus was room air pumped through a flask of water (RH > 75%) or silica gel (RH < 10%) and passed to a custom switching device with a solenoid valve for each stream. When activated, this device would pass one stream to vent while it passed the other to the preparation. A timing device controlled the solenoid valves and provided stimuli of known durations. A brief electrical pulse was introduced into the spike train when the solenoid valves were switched to indicate stimulus onset and offset.

Air flow was measured using a soap film flow-meter. The average flow rates were 0.88 liters per minute (moist, SE = 0.005) and 0.87 liters per minute (dry, SE = 0.004). A t-test showed that there was no significant difference between the flow rates of the two air streams.

**Figure 5.**
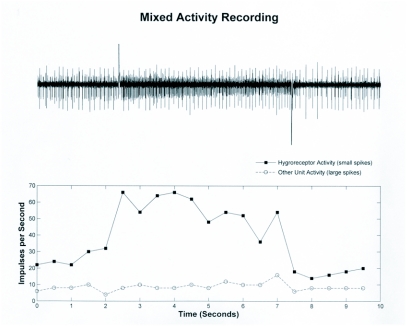
Mixed activity recording. A ten second recording from two units, one of which is a hygroreceptor. The large artifacts in the trace indicate the switch from dry to moist air stimulus (positive going) and then back to dry air (negative going). The graph shows the activity of the units in 0.5 second bins.

#### Data analysis

Recordings were exported from STA into MATLAB for spike identification and analysis.

## Results

### Hygropreference

Larvae were monitored in a linear hygropreference chamber to test the hypotheses that they would spend more time in regions of higher relative humidity, and that this behavior would be influenced by their hydration state. Control larvae did not show any preference for a specific relative humidity at any temperature tested (Figure 2), but did display a preference for the two end chambers of the linear arena compared to the three more central chambers. Desiccated larvae preferred the most humid chamber at all three temperatures (Figure 2). This preference was significant when compared with the corresponding control (25 °C: p < 0.01, 30 °C and 34 °C: p < 0.001). No significant effect of temperature on preference was observed. In contrast, desiccated larvae whose antennae were coated with either of the wax mixtures failed to show a preference for the humid chamber (Figure 3A). They did, however, show an end preference similar to the control larvae. Desiccated larvae whose antennae were surgically removed showed neither the preference for the humid chamber nor the ends of the arena (Figure 3B).

**Table 2:**
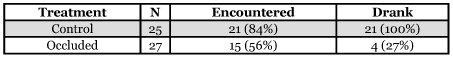
Water drinking behavior. The data show the number of larvae with normal or occluded antennae that encountered and drank liquid water. Numbers in parenthesis are the percent of total larvae that encountered the water drop (third column), or the percent of encountering larvae that drank (fourth column).

### Liquid water orientation

To examine the ability of larvae to detect and respond to liquid water, we tested them in the small Petri dish with wax floor containing a drop of water. All larvae moved apparently randomly around and across the arena floor and there appeared to be no difference in the amount of activity between treatment groups. Control larvae normally encountered the water drop (84% of those tested) and always drank the water when it was encountered. Occluded larvae, although similarly active within the arena, encountered the water drop less frequently (55.6% of those tested), and only 27% of these drank from it (Table 2).

**Figure 6.**
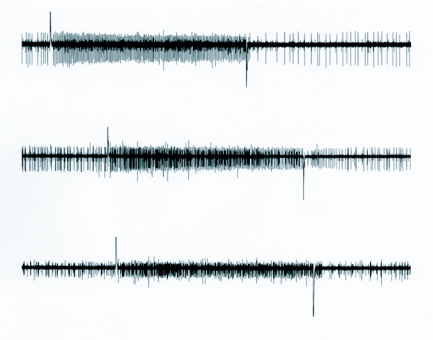
Inter-individual variability. Ten-second recordings from 3 different animals illustrate some of the observed variability. Note the post-stimulus inhibition, particularly in the first two traces.

Electrophysiological recordings confirmed the presence of a responding unit (presumably a sensory neuron) that responds to changes in relative humidity. This neuron is located in the vicinity of the medial basiconic sensilla of the second antennal segment. The best recordings were achieved by entering the cuticle just at the base of this structure. Within this region there are several units that fire either spontaneously or in response to movement of the electrode. Near the hygroreceptive cell is another cell that fires spontaneously at a regular rate. This cell is consistently observed in most preparations and was used as a landmark while searching for the hygroreceptive cell.

In some instances, fortunate placement of the electrode permitted the recording of only the hygroreceptive cell that was silent in dry air (Figure 4). However it was more common to record from multiple cells at a time, and the hygroreceptive neurons showed some level of activity in a dry air stream (Figure 5). Actual spike frequency and activity in dry air were variable between animals, although an increase in activity occurred in all recordable hygroreceptive cells upon exposure to a moist air stream (e.g. Figure 6). Adaptation in general was slow but was observed in at least three preparations over a 30 second period (data not shown).

Many if not all other areas of the antenna were probed with this type of electrode, but no other neurons sensitive to relative humidity were found.

## Discussion

We hypothesized that larval *M. sexta* prefer humid environments and that they would use relative humidity as a cue to make locomotory decisions. The data indicate that *M. sexta* larvae can detect relative humidity in their environment and will seek areas of higher relative humidity under certain conditions. This preference is highly dependent on internal hydration state. Control larvae showed no preference for a specific relative humidity, while desiccated larvae showed a significant preference for the high humidity chamber.

The hypothesis that temperature would affect this behavior was not supported. Neither desiccated nor control larvae altered their hygropreference behavior with temperature. While increased environmental temperature will certainly increase evaporative water loss, the larvae may only monitor internal hydration state.

The data suggest that instead of attempting to prevent desiccation stress, the larvae take corrective measures to reduce water loss by seeking regions of high humidity only after they have become stressed. This strategy may reflect a balance between the need for hydration homeostasis and the need to forage for food. When a larva is not under desiccative stress, the need to forage likely outweighs the risks of prospective exposure to desiccative stress. Thus under most conditions, proactive hygropreference would not be expected.

On the other hand, proactive hygropreference behavior may occur when preparing to molt. When molting, larvae become quiescent for one to two days and would likely risk desiccation if exposed on the upper leaf surface. This is consistent with greenhouse observations where most larvae molt on the underside of the host plant's leaves. By moving to the underside of the leaf the larva places itself into an area of locally higher relative humidity, thus reducing desiccative stress.

The antennae appear to be required for proper hygropreference behavior and likely house the sensory neurons responsible for this behavior. Ablation or occlusion of the antennae eliminated the preference for high humidity. These behavioral results are consistent with the electrophysiological confirmation that there is a sensory neuron, located on the second antennal segment, that responds differentially to wet and dry air streams.

The presence of neurons on the antenna of larval *M. sexta* that respond to changes in relative humidity was briefly reported previously by Dethier and Schoonhoven (1968). The electrophysiological experiments described here were conducted to confirm the presence of these sensory neurons and to further characterize their behavior. Recordings from the region near the medial basiconic sensillum of the second antennal segment revealed the presence of at least one neuron that responds differentially to wet and dry air streams. Recording in other regions of the antennae yielded no other hygroreceptive neurons. Thus, it seems likely that the hygroreceptors of the medial basiconic sensilla are necessary and sufficient for mediating hygropreference behavior.

To extend this conclusion to a related behavior, the ability of the larvae to orient towards and drink liquid water was examined. In the Petri dish arenas, control larvae had little problem finding and drinking the water. The hypothesis that the same antennal hygroreceptors would be required for this behavior as well was confirmed. Indeed, larvae with occluded antennae located the water less frequently and rarely drank from it when they did encounter it. These results further support the hypothesis that the antennal hygroreceptors are essential for water related behavior in larval *M. sexta.*

Drinking behavior was easy to identify after a short period of observation. Typically a control larva would orient towards the water drop within the first ten minutes of the trial. Upon making contact with the water drop with its mouthparts and antennae, the larva would cease locomotion, consume it, and resume movement within the arena. Most of the occluded larvae seemed to wander randomly within the arena. Those that encountered the water drop usually walked through it and did not arrest their movement upon contacting it.

These results are consistent with preliminary experiments in which a larva was held in hand and a drop of water from a pipette was placed on its mouthparts and antennae. Control larvae would immediately begin moving their mandibles and drinking. Occluded larvae would not respond to the water drop. Neither group of larvae responded when prodded with only the pipette indicating that it was the presence of the water to which they were responding, not a mechanical stimulus.

Larvae with ablated or occluded antennae still had all of their chemosensory mouthparts unobstructed. Post-experimental microscopic observations verified that the maxillary palpi, the sensilla styloconica, and the epipharynx were all intact and uncoated with wax. Thus we conclude that the observed drinking and hygropreference behaviors are mediated only through the antennae.

Since animals that live in arid conditions are often at high risk for desiccation, being able to make use of available water resources could be a significant factor in their survival. We have demonstrated that larval *M. sexta* can detect both relative humidity and liquid water via their antennae. This information, coupled with internal hydration state information, is used in locomotory decision making. Such decisions likely form the basis of a behavioral strategy for maintenance of hydration homeostasis. Coupled with possible physiological mechanisms for desiccation prevention (Woods & Bernays 2000), this strategy helps explain how larval *M. sexta* are able to thrive in arid habitats.
